# Regulation of cellular sphingosine-1-phosphate by sphingosine kinase 1 and sphingosine-1-phopshate lyase determines chemotherapy resistance in gastroesophageal cancer

**DOI:** 10.1186/s12885-015-1718-7

**Published:** 2015-10-22

**Authors:** Kasia Matula, Elaina Collie-Duguid, Graeme Murray, Khyati Parikh, Heike Grabsch, Patrick Tan, Salina Lalwani, Roberta Garau, Yuhan Ong, Gillian Bain, Asa-Dahle Smith, Gordon Urquhart, Jacek Bielawski, Michael Finnegan, Russell Petty

**Affiliations:** 1 School of Medicine and Dentistry, University of Aberdeen, Aberdeen Royal Infirmary, Foresterhill Healthcare Campus, Foresterhill, Aberdeen, AB25 2ZG Scotland UK; 2 Department of Pathology, University of Aberdeen, Aberdeen Royal Infirmary, Foresterhill Healthcare Campus, Foresterhill, Aberdeen, AB25 2ZG Scotland UK; 3 Department of Gastroenterology, University of Aberdeen, Aberdeen Royal Infirmary, Foresterhill Healthcare Campus, Foresterhill, Aberdeen, AB25 2ZG Scotland United Kingdom; 4 Department of Oncology, University of Aberdeen, Aberdeen Royal Infirmary, Foresterhill Healthcare Campus, Foresterhill, Aberdeen, AB25 2ZG Scotland UK; 5 Department of Pathology, Maastricht University Medical Center, Maastricht, The Netherlands; 6 Cancer Science Institute of Singapore National University of Singapore, Singapore, Singapore; 7 Department of Biochemistry and Molecular Biology, Medical University of South Carolina, Charleston, SC USA; 8 Division of Cancer Research, School of Medicine, University of Dundee, Mailbox 4, Level 7 Ninewells Hospital and Medical School, Dundee, DD1 9SY Scotland UK

**Keywords:** Gastroesophageal cancer, Chemoresistance, Sphingosine-1-phosphate, Sphingosine kinase 1, Sphingosine-1-phopshate lyase

## Abstract

**Background:**

Resistance to chemotherapy is common in gastroesophageal cancer. Mechanisms of resistance are incompletely characterised and there are no predictive biomarkers in clinical practice for cytotoxic drugs. We used new cell line models to characterise novel chemotherapy resistance mechanisms and validated them in tumour specimens to identify new targets and biomarkers for gastroesophageal cancer.

**Methods:**

Cell lines were selected for resistance to oxaliplatin, cisplatin and docetaxel and gene expression examined using Affymetrix Exon 1.0 ST arrays. Leads were validated by qRT-PCR and HPLC of tumour metabolites. Protein expression and pharmacological inhibition of lead target SPHK1 was evaluated in independent cell lines, and by immunohistochemistry in gastroesophageal cancer patients.

**Results:**

Genes with differential expression in drug resistant cell lines compared to the parental cell line they were derived from, were identified for each drug resistant cell line. Biological pathway analysis of these gene lists, identified over-represented pathways, and only 3 pathways - lysosome, sphingolipid metabolism and p53 signalling- were identified as over-represented in these lists for all three cytotoxic drugs investigated. The majority of genes differentially expressed in chemoresistant cell lines from these pathways, were involved in metabolism of glycosphingolipids and sphingolipids in lysosomal compartments suggesting that sphingolipids might be important mediators of cytotoxic drug resistance in gastroeosphageal cancers . On further investigation, we found that drug resistance (IC50) was correlated with increased sphingosine kinase 1(SPHK1) mRNA and also with decreased sphingosine-1-phosphate lysase 1(SGPL1) mRNA. SPHK1 and SGPL1 gene expression were inversely correlated. SPHK1:SGPL1 ratio correlated with increased cellular sphingosine-1-phosphate (S1P), and S1P correlated with drug resistance (IC50). High SPHK1 protein correlated with resistance to cisplatin (IC50) in an independent gastric cancer cell line panel and with survival of patients treated with chemotherapy prior to surgery but not in patients treated with surgery alone. Safingol a SPHK1 inhibitor, was cytotoxic as a single agent and acted synergistically with cisplatin in gastric cancer cell lines.

**Conclusion:**

Agents that inhibit SPHK1 or S1P could overcome cytotoxic drug resistance in gastroesophageal cancer. There are several agents in early phase human trials including Safingol that could be combined with chemotherapy or used in patients progressing after chemotherapy.

**Electronic supplementary material:**

The online version of this article (doi:10.1186/s12885-015-1718-7) contains supplementary material, which is available to authorized users.

## Background

The clinical outcomes for gastroesophageal cancer are poor. One year survival is only 40–50 % and 5 year survival 10–20 % [1]. At the time of clinical diagnosis only 30–40 % patients have loco-regionally confined disease that is amenable to potentially curative therapy and the majority of patients relapse systemically after such treatment [1].

These outcomes are largely the consequence of systemic dissemination at a very early stage and indicate the importance of systemic therapies in disease management [2, 3]. Accordingly, cytotoxic chemotherapy has value as neo-adjuvant, adjuvant and palliative treatment [2–4]. Cisplatin, oxaliplatin and docetaxel are amongst the most active cytotoxics and key components of combination chemotherapy regimens [2, 5]. Nevertheless, resistance to cytotoxic drugs is common and severely limits the effectiveness of these treatments by resulting in the delivery of ineffective and toxic therapy.

Accordingly, identification of predictive biomarkers for chemotherapy in gastroesophageal cancer are urgently needed in clinical practice and would enable a stratified approach to treatment selection, and optimise clinical and cost effectiveness. Despite extensive investigation there are no predictive biomarkers for chemotherapy that are recommended for clinical use in gastroesophageal cancer. More recently the use of global molecular analysis tools such as gene expression profiling, array-CGH, exome and whole genome sequencing, has provided more promising leads for predictive biomarkers for chemotherapy in gastroesophageal cancer [6, 7].

Predictive biomarkers for chemotherapy resistance may also have value as therapeutic targets for agents that would combine effectively with cytotoxic drugs. A clinical proof of principle for the safety, tolerability and effectiveness of combining targeted agents with chemotherapy as part of a biomarker directed stratified therapy approach, has been demonstrated recently in gastroesophageal adenocarcinoma, combining trastuzumab with cisplatin and 5FU in patients whose tumours are HER 2 positive [8]. However only 10–15 % of gastroesophageal adenocarcinomas are HER2 positive and the identification of clinically effective targeted agents has proven challenging in gastroesophageal cancer, with Phase III trials evaluating the addition of targeted therapies against Epidermal Growth Factor Receptor (EGFR), Vascular Endothelial Growth Factor (VEGF), Mammalian Target of Rapamycin (mTOR) Mamalian mTOR, to cytotoxic chemotherapy, not demonstrating any benefit [9–12], and there are no targeted therapy options at all for squamous cell carcinoma of the esophagus. More recently, the addition of the VEGFR-2 targeting agent Ramicurumab to paclitaxel chemotherapy has been shown to be beneficial in a phase III randomised controlled trial, but as yet there are no predictive biomarkers for Ramicurimab, which is likely to significantly limit the cost effectiveness of this treatment [13]. Overall, there is a clear ongoing clinical need to identify further new targets and biomarker combinations for gastroesophageal cancer, in particular those which might combine effectively with cytotoxic chemotherapy.

In order to address this we utilised gastroeosphageal cancer cell lines selected for resistance to cisplatin, oxaliplatin and docetaxel as models for the identification of new markers of drug resistance and candidate novel therapeutic targets. Such models have been widely used and have provided new insights into mechanisms of drug action and resistance, but translation from such studies to clinically useful targets or biomarkers has been more limited [14]. In light of this, and the more recent demonstration of the usefulness of global molecular profiling tools with gastroesophageal cancer cell line models to identify predictive markers and targets [6, 7], we used global gene expression profiling on our cytotoxic resistant cell lines to identify lead molecules for further investigation. To further determine their clinical utility as predictive biomarkers and/or novel therapeutic targets leads were validated by quantitative real-time polymerase chain reaction (qRT-PCR), assay of relevant tumour metabolites in key biological pathways, pharmacological inhibition of an identified target, and evaluation of predictive and prognostic value in an independent panel of gastric cancer cell lines and tumour tissues from gastroesophageal cancer patients.

## Methods

### Cell Lines and cell culture

Human esophageal squamous carcinoma (OE21), adenocarcinoma of oesophagus (OE33), and adenocarcinoma of gastric cardia (AGS) cancer cell lines were obtained from the European Collection of Animal Cell Culture (Centre for Applied Microbiology and Research, Salisbury, UK). OE21, OE33 and AGS cell lines were cultured and maintained in RPMI - 1640 medium, supplemented with 10 % (v/v) foetal calf serum and 1 % (v/v) penicillin/streptomycin (100 000 U/l penicillin, 100 mg/l streptomycin). Gastric cancer cell lines Kato III, NCI-N87 and Hs746T were obtained from American Type Culture Collection, Manassas, VA, USA), and cultured as recommended by the supplier. Gastric cancer cell lines AZ521, Fu97, IM95, Ist1, MKN1, MKN45, MKN7,MKN28, MKN45 and TMK1 cells were obtained from the Japanese Collection of Research Bioresources and cultured as recommended . The SCH gastric cancer cells were a gift from Yoshiaki Ito (Institute of Molecular and Cell Biology, Singapore) and grown in RPMI supplemented with 10 % (v/v) foetal calf serum and 1 % (v/v) penicillin/streptomycin (100 000 U/l penicillin, 100 mg/l streptomycin). The gastric cancer cell lines YCC1, YCC3, YCC6, YCC7, YCC10, YCC11and YCC16 cells were a gift from Sun-Young Rha (Yonsei CancerCenter, Seoul, South Korea) and were grown in minimum essential medium supplemented with 10 % fetal bovine serum, 100Uml1penicillin, 100Uml1 streptomycin and 2 mmol l1L-glutamine (Invitrogen, Carlsbad, CA, USA). All cells were cultured at 37 °C in a humidified atmosphere containing 5 % carbon dioxide. All cell lines were tested and authenticated by the cell line bank provider (ECACC, ATCC, JCRB) or the originating institution (YCC and SCH) by several methods including Short Tandem Repeat profiling and/or cytogenetics(and cells utilised within 6 months of receipt). Prior to this study, we re-authenticated the cell lines by comparing their genome-wide gene expression profiles (Affymetrix Exon 1.0 ST Arrays (1 084 639 exons and over 300 000 transcript clusters on each oligonucleotide microarray; www.affymetrix.com) and/or mutational profiles, and/or their genome-wide copy number (Agilent Human Genome244A CGH Microarrays, Agilent Technologies, Santa Clara, CA) to that in public databases and published literature. Ethical approval was not required for the use of the cell lines in this investigation.

### Drugs and reagents

Oxaliplatin, cisplatin, docetaxel and 3-((4, 5-dimethylthiazol-2-yl)-2, 5-diphenyltetrazolium bromide,MTT) solutions were obtained from Sigma-Aldrich(UK). RPMI-1640(GlutaMAX) culture medium from GIBCO(BRL); Foetal bovine serum from Thermo Scientific; Penicillin/streptomycin were obtained from Sigma-Aldrich (UK). All reagents were molecular biology grade unless otherwise stated.

### Cell Viability Assays

MTT and MTS assay were used as indicated to assess cytotoxicity. Assays were performed on 96- well plates with complete media alone (no cells) as a background control, and blank and vehicle controls included on each plate. Unless otherwise stated, all measurements were performed in triplicate independent experiments with triplicate data points within an assay. Paired parental and resistant daughter lines were tested in parallel on the same plate. The MTT assay was performed as previously described [15] with absorbance measured at 570 and 690 nm using Gen 5 v.2 software on a multi-well plate reade (BioTek, Synergy HT). MTS assays were performed using a commercially available kit(MTS kit; Promega, Madison, WI, USA), according to the manufacturer’s instructions. In all cases cell lines were seeded in 100 μl of media in a 96-well plate and left to adhere for 24 h, 100 μl of drug diluted in media was added and incubated for 72 h at 37 °C and 5 % CO2 and absorbance measured using an EnVision2104 multi-label plate reader (Perkin Elmer, Turku, Finland) at 490 nm. A dose curve was fitted and IC50 values representing the drug concentration required to elicit a 50 % growth inhibition compared to vehicle control were calculated in Prismv6 software (GraphPad PRISM v.5.02, La Jolla, CA, USA).

### Generation of resistant cell lines

OE21, OE33 and AGS cell lines were selected for progressive resistance to oxaliplatin, cisplatin and docetaxe las described previously [15]. Briefly, selection began at a drug dose that was 20 fold less than the half maximal inhibitory concentration (IC50) concentration. Cells were grown at the same drug concentration over 4 passages and then cell viability tests performed. Drug concentrations were increased 2 to 4 - fold until the IC50 daughter/IC50 parental ≥ 3. The panel of drug resistant cell lines generated in this way were AGS_CIS5_, AGS_OX8_, AGS_DOC6_, OE33_CIS4_, OE33_OX4_ and OE21_OX4_ with the subscript denoting the drug and final concentration of drug (μM) that cells were exposed to. Changes in IC50 during generation of drug resistant cell lines are presented in Additional file 1: Additional information 1.

### Gene expression Profiling

Gene expression was assessed using the Affymetrix Exon 1.0 ST Arrays (1 084 639 exons and over 300 000 transcript clusters on each oligonucleotide microarray; www.affymetrix.com). Details of RNA extraction, sample preparation and quality control are described in Additional file 1: Additional Information 2. Gene expression profiling data is available in MIAME compliant format in Array Express (www.ebi.ac.uk/arrayexpress) accession number E-MTAB-2860.

### Analysis of gene expression data

Gene expression data was analysed using GeneSpring v.11.1 (Agilent, Wokingham, UK) and DAVID v6.7 for pathway analysis (NIH, Bethesda, MD, USA) [16]. Core probe sets on the Human Exon 1.0 ST array were processed using the RMA16 algorithm (Affymetrix, Santa Clara, CA, USA) that employs quantile normalisation of log2 transformed data. Data were transformed to the median of all samples. Further details of gene expression analysis and details for pathway analysis are described in Results and Additional file 1: Additional information 3.

### Quantitative real-time PCR

Roche LightCycler 480 master mix (Roche Diagnostics GmbH, Mannheim, Germany) was used, with conditions: 95 °C for 5 min followed by 45 cycles of 95 °C for 10 s and 60 °C for 15 s. The amplified fluorescent signal was detected and relative quantification was assessed with LightCycler 480 SW v 1.5(Roche Diagnostics). Gene expression was normalised to GAPDH and changes in expression measured relative to the parental line as a control. PCR primer sequences used (Sigma - Genosys, Haverhill, UK) are in Additional file 1: Additional Information 4. For each gene, all experiments were repeated in triplicate using RNA extracted from three independent samples.

### Analysis of Spingosine-1-Phosphate

Analysis and quantification of sphingosine-1-phosphate from cell lines, including the use and preparation of all internal standards and reagents was using the high performance liquid chromatography-tandem mass spectrometry (HPLC-MS/MS) method as described by Bielawski et al. [17]. Further details of equipment used and preparation of cell pellets and lipid extraction are provided in Additional file 1: Additional information 5. Analysis was performed in duplicate and to limit inter-assay variability each WT line was analysed in parallel with each drug resistant daughter line. The level of S1P was determined in pmol/sample, with samples normalized to total phosphorus content.

### Patients

Formalin fixed paraffin embedded (FFPE) tumour tissues were obtained from patients with esophageal or gastric cancers who underwent surgical resection at Aberdeen Royal infirmary between 2004 and 2009. 36/67 patients received neo-adjuvant chemotherapy with 3 cycles of Epirubicin, Cisplatin and Capecitabine prior to surgery. Clinico-pathological features of patients are detailed in Table 1 and further details of treatment are provided in Additional file 1: Additional Information 6. The use of these tissues was approved by the North of Scotland research ethics committee and proceeded with informed consent.

**Table 1:** Clinical details of patients treated with surgical resection of gastroesophageal cancers

Variable	Score	Neo-adjuvant Chemotherapy	No Neo-adjuvant Chemotherapy	*P* value*
Age	>Median	9	15	0.073
	< Median	27	16	
Histology	Adenocarcinoma	31	24	0.180
	Squamous	5	7	
Stage	I	11	3	0.109
	II	10	12	
	III	15	16	
Site	Oesophagus	30	25	0.755
	(includes Siewert Type I and II junctional)			
		6	6	
	Gastric			
	(includes Siewert Type III junctional)			
Circumferential Resection Margins	Positive	6	11	0.378
	Negative	30	20	

There was no significant difference in clinic-pathological characteristics between patients who did and did not receive neo-adjuvant chemotherapy prior to surgery. SPHK1 immunohistochemistry was performed on this cohort as described. *χ^2^ test. Two-sided p value

### Immunohistochemistry

Representative 4 μm sections of FFPE tumours or cell line pellets were mounted onto glass slides rehydrated following a standard protocol. Individual cell line pellets were prepared from cultured cell lines harvested and fixed in 4 % paraformaldehyde, and further processed for paraffin embedding as described in [18] Antigen retrieval was performed by microwaving in 10 mM citrate (pH 6.0) for 20 min. SPHK1 (1:60, tumours, 1:400 cell lines) rabbit polyclonal antibody (Abgent, CA, USA) was used with an autostainer (Dakocytomation, Glostrup, Denmark) and the CSAII detection system according to the manufacturer's instructions. All sections were double scored by two independent investigators who were blinded to the clinical data. Overall, more than 90 % agreement in scoring was observed. Scoring discrepancies were resolved by examination of sections at a double-headed microscope.

### Statistical analysis

All other statistical analyses including survival analysis were performed using PASW statistics v20 (IBM Corporation, Armonk, NY, USA). Kaplan–Meier survival curves with log rank test and cox proportional hazards analysis were used for survival analysis and survival time was calculated from date of histological diagnosis until date of death. Fisher’s exact test or Pearson chi-square was used for the assessment of categorical variables and Student’s *t*-test, one way- ANOVA, 2-way ANOVA with Sidlak post-hoc test for continuous variables. All reported P-values are two sided and p < 0.05 was considered statistically significant. Combination Index to quantify synergy between cisplatin and safingol was calculated using Compusyn(Combosyn, Paramus, NJ).

## Results

### Lysosomal and sphingolipid metabolism genes are differentially expressed in drug resistant cancer cell lines

**Fig. 1:**
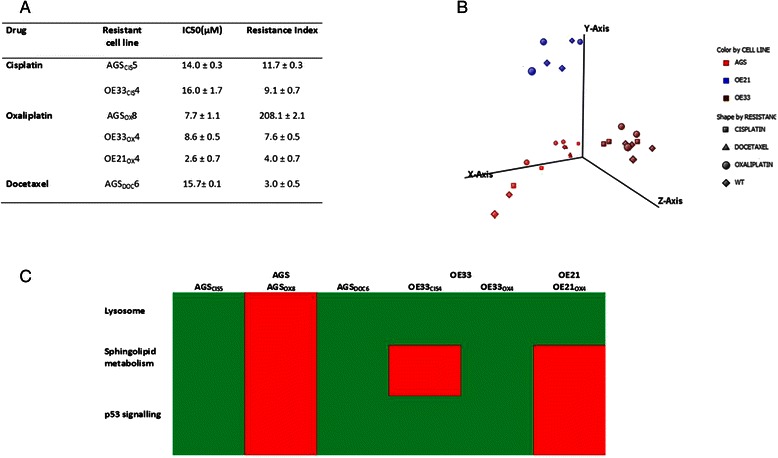
Development and characterisation by gene expression profiling of cytotoxic drug resistant gastroesophageal cancer cell lines. **a** Drug resistant cell lines used in this study (see also Additional file 1). **b** Principle component analysis of drug resistant cell lines using 16939 genes expressed in at least 1 cell line (threshold of expression ≥ 20th percentile) with 3 independent replicates per cell line from three different passages using Affymetrix Exon 1.0ST microarrays (see also Additional File 1: Additional information 2). **c** Only 3 pathways, namely the lysosome, sphingolipid metabolism and p53 signalling were identified as over-represented in gene set enrichment analysis of genes significantly differentially expressed for all 3 cytotoxic drugs compared to sensitive parental lines and in each case they were also identified in at least 2 cell lineages (DAVID v6.7 for biological pathway mapping and gene set enrichment analysis (EASE score, modified Fisher exact p < 0.05 [16]), Paired *t*-test with Benjamini and Hochberg correction for multiple testing (corrected P <0.05) to derive the differentially expressed gene set, green = over represented red = not over-represented. See also Additional file 1: Additional information 7

Gene expression was performed using RNA isolated from AGS, AGSCIS5, AGSOX8, AGSDOC6, OE33, OE33CIS4, OE33OX4, OE21, OE21OX4 (Fig. 1a), with 3 independent replicates per cell line from three different passages. Core gene sets were analysed and using threshold of expression ≥ 20th percentile there were 16939 out of 17881 genes expressed in at least 1 cell line. Principle component analysis using these 16939 genes revealed clustering according to cell line rather than drug resistance or histological subtype (Fig. 1b). Statistical filtering (Unpaired *t*-test with Benjamini and Hochberg MTC corrected *p* < 0.05) of these 16939 genes was performed on each pair of drug resistant versus parental cell line. This analysis identified differentially expressed genes for drug resistant gastric adenocarcinoma [AGSCIS5 (*n* = 1298), AGSOX8 (*n* = 466), AGSDOC6 (n = 2251)], esophageal adenocarcinoma [OE33OX4 (n = 2107), OE33CIS4 (*n* = 2613)] and esophageal squamous cell carcinoma [OE21OX4 (*n* = 859)] cell lines compared to the sensitive parental line.

**Fig. 2:**
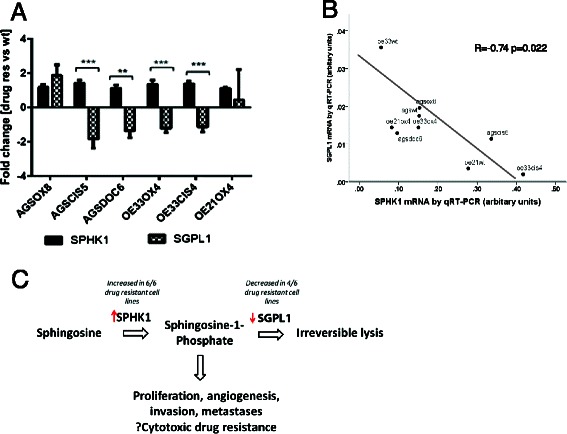
SPHK1 and SGPL1 expression in cytotoxic drug resistant gastroesophageal cancer cell lines. **a** All drug resistant cell lines showed increased SPHK1 mRNA expression relative to parental wild type line, and 4 out of 6 also showed decreased SGPL1 mRNA. Data shown is mean (+/- SEM) from 3 independent replicates per cell line from three different passages using Affymetrix Exon 1.0ST microarrays and validated by qRT-PCR (see Additional file 1: Additional information 2 and 3). *** *p* < 0.01 ** *p* < 0.05 (Student’s *t*-test)**. b** Inverse correlation between SPHK1 and SGPL1 mRNA levels (*R* = -0.740, *p* = 0.022). Data shown is mean for all drug resistant cell lines and parental wild type lines, 3 independent replicates per cell line from three different passages measured by qRT-PCR (see also Additional file 1: Additional information 5). **c** Hypothesis of increased SPHK1 and decreased SGPL1 leading to increased S1P in gastro-oeosphageal cancer, promoting cell survival and hence cytotoxic drug resistance

Gene enrichment analysis (DAVID v6.7, *p* < 0.05) identified pathways that were over-represented among each of these gene lists (Additional file 1: Additional information 7). A number of pathways reported as being important in cisplatin,oxaliplatin and docetaxel drug resistance for example p53 signalling, base excision repair and DNA replication were identified. Only 3 common ontologies (biological pathways/cell component), namely the lysosome, sphingolipid metabolism and p53 signalling, were identified for all 3 cytotoxic drugs. For each drug, at least 2 of the gastroesophageal cancer cell lineages had significant enrichment of these biological networks in the drug resistance gene set (Fig. 1c). Accordingly, these pathways were selected for further investigation as potential novel mechanisms of cytotoxic drug resistance in gastroesophageal cancer. The lysosome was identified in the analysis for all 3 cell lineages and all 3 cytotoxic drugs. A comprehensive analysis of the published literature and databases revealed that the protein products of the majority of the genes identified as differentially expressed in the resistant lines in the pathways from the gene enrichment analysis, were involved in metabolism of glycosphingolipids and sphingolipids in lysosomal compartments. This was reflected in identification of sphingolipid metabolism in gene set enrichment analysis. These data suggested that sphingolipids might be important mediators of cytotoxic drug resistance in gastroeosphageal cancers. In support of this hypothesis, our gene expression profiling data identified increased expression of sphingosine- kinase 1 (SPHK1), required for metabolism of sphingosine to sphingosine-1-phosphate (S1P), in all resistant lines and decreased expression of sphingosine -1 Phosphate lysase (SGPL1), catalysing irreversible lysis of S1P, in 4 out of 6 of the resistant cell lines (AGS_CIS5_, AGS_DOC6_, OE33_OX4_, OE33_CIS4_) compared to their parental wild type lines (Fig. 2a). Furthermore, there was a significant inverse correlation observed between SPHK1 mRNA expression and SGPL1 mRNA expression in all the gastroesophageal cancer the cell lines (*R* = -0.740, *p* = 0.022, Fig. 2b).

SPHK1 and SGPL1 mRNA levels measured on microarrays were validated by qRT-PCR, with strong correlations between gene expression measured with each assay (SPHK1 *R* = 0.731, *p* = 0.005, SGPL1 *R* = 0.867, *p* = 0.002).

Many previous investigations have identified SPHK1 as overexpressed in several cancer types including gastric adenocarcinoma and associated with increased stage and poor survival [19–21]. In addition, preclinical investigations in cancer and non-cancer cells demonstrate that increased SPHK1 is associated with increased production of sphingosine-1-phosphate (S1P) in cancer cells and S1P promotes cell proliferation and angiogenesis, and inhibits cell death [22–29]. SPHK1 activity and levels of S1-P have been demonstrated to be involved in resistance to cytotoxic and targeted agents in a variety of cancer types, although not in esophageal or gastric cancer drug resistance [30–36]. SGPL1 is responsible for the irreversible cleavage of S1P into hexadecenal and ethanolamine phosphate, but there has been little investigation of SGPL1 in human cancers. Recently, in prostate cancer, an inverse relationship between expression of SPHK1 and SGPL1 was noted and down regulation of SGPL1 increased production of S1P and was associated with resistance to docetaxel [37].

Therefore it seemed biologically plausible to hypothesise, based upon the analysis of our gene expression data, that in gastroesophageal cancer increased expression of SPHK1, often associated with decreased expression of SGPL1, would lead to increased S1P potentially a pathogenic mechanism in gastroesophageal cancer cells, which would also lead to cytotoxic drug resistance (Fig. 2c).

### Ratio of SPHK1:SGPL1 mRNA correlates with cellular S-1-P in gastroesophageal cancer cell lines

**Fig. 3:**
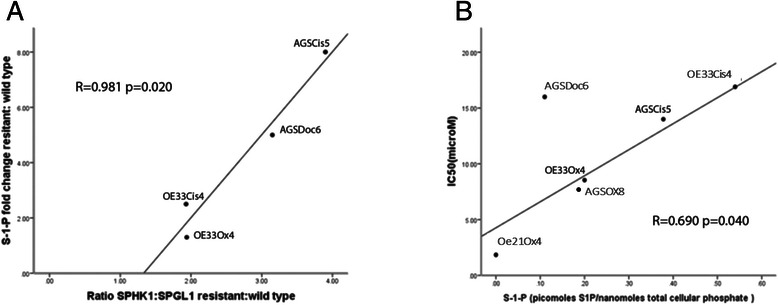
Relationship between SPHK1 and SGPL1 expression and S1P and cisplatin resistance in gastroesophageal cancer cell lines. **a** In drug resistant cell lines that demonstrate increased SPHK1 together with decreased SGPL1 (AGS_CIS5_, AGS_DOC6_,OE33_OX4_, OE33_CIS4_), the fold change in the ratio of SPHK1:SGPL1 mRNA correlates with observed increase in cellular S1P in drug resistant cell lines relative to the respective parental cell lines (*R* = 0.981, *p* = 0.020). Data shown is mean for 3 independent replicates per cell line from three different passages measured by qRT-PCR (see also Additional file 1: Additional information 5). **b** In the drug resistant cell lines (Fig. 1a), cellular S1P correlates with IC50 to cisplatin, oxaliplatin and docetaxel, respectively (*R* = 0.690, *p* = 0.040). IC50 data determined by MTT assay with each data point in each cell line measured in triplicate with 3 independent replicate experiments. S1P measured using high performance liquid chromatography-tandem mass spectrometry as described in the text, mean value from duplicate assays

In order to test this hypothesis we examined the relationship between the cellular levels of S1P and the ratio of SPHK1 and SGPL1 mRNA expression and drug resistance in the 4 drug resistant cell lines that demonstrated increased SPHK1 together with decreased SGPL1 - AGS_CIS5_, AGS_DOC6_,OE33_OX4_, OE33_CIS4_. There was a strong correlation between the SPHK1:SGPL1 mRNA ratio in the drug resistant cell lines and the increase in S1P observed in the drug resistant cell lines compared to the relevant parental wild type line (*R* = 0.981, *p* = 0.020, Fig. 3a).

### Cellular S-1-P correlates with IC50 in gastroesophageal cell lines

We further investigated the relationship between drug resistance and cellular levels of the sphingosine metabolite, S1P. Increased cellular S1P levels correlated with increased IC50 in drug resistant lines (*R* = 0.690, *p* = 0.040, Fig. 3b). This relationship between cellular S1P and IC50 was observed across oxaliplatin, cisplatin and docetaxel resistant cell lines.

### SPHK1 mRNA correlates with SPHK1 protein expression in gastroesophageal cell lines

**Fig. 4:**
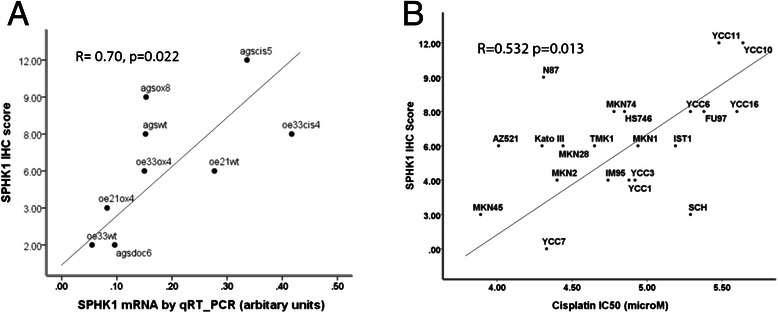
Relationship between SPHK1 protein expression and cisplatin resistance in gastroesophageal cancer cell lines. **a** SPHK1 protein expression determined by semi-quantitative immunohistochemistry Q-score(see text) correlates with SPHK1 mRNA expression in drug resistant gastroesophageal cell lines(mean for all drug resistant cell lines and parental wild type lines, 3 independent replicates per cell line from three different passages measured by qRT-PCR (see also Additional file 1: Additional information 5). (*R* = 0.70, *p* = 0.022). **b** SPHK1 protein expression determined by semi-quantitative immunohistochemistry Q-score(see text) with IC50 for cisplatin in an independent panel of gastric cancer cell lines. (*R* = 0.532, *p* = 0.013)

Formalin fixed paraffin embedded individual cell line pellets were prepared from cultured cells and SPHK1 protein expression was measured by immunohstochemistry (IHC) using a pre-determined semi-quantitative Quick-score. SPHK1 IHC Quick-score = intensity x proportion: intensity scored as 0 = negative, 1 = weak, 2 = moderate and 3 = strong SPHK1 staining in tumour cells; positive proportion scored as 0 = 0 %, 1 = 1-10 %, 2 = 11-50 %, 3 = 51-70 %, and 4 = > 70 % tumour cells positive for SPHK1 staining. In the parental and drug resistant cell lines a strong correlation between SPHK1 mRNA expression and SPHK1 protein expression was observed (*R* = 0.070 *p* = 0.022 Fig. 4a).

### SPHK1 protein expression in an independent panel of gastric cancer cell lines correlates with resistance to cisplatin

We examined the relationship between SPHK1 protein expression measured by IHC, and cisplatin resistance in an independent panel of 21 gastric cancer cell lines. The independent panel of 22 gastric cancer cell lines comprised: Kato III, NCI-N87, Hs746T,AZ521, Fu97, IM95, Ist1, MKN1, MKN45, MKN74,MKN28, MKN45,TMK1,SCH,YCC1, YCC3, YCC6, YCC7, YCC10, YCC11 and YCC16. There was a significant relationship between SPHK1 protein expression and IC50 for cisplatin (*R* = 0.532 *p* = 0.013, Fig. 4b).

### High SPHK1 protein expression is associated with poor survival in Gastroesophageal cancer patients treated with chemotherapy

**Fig. 5:**
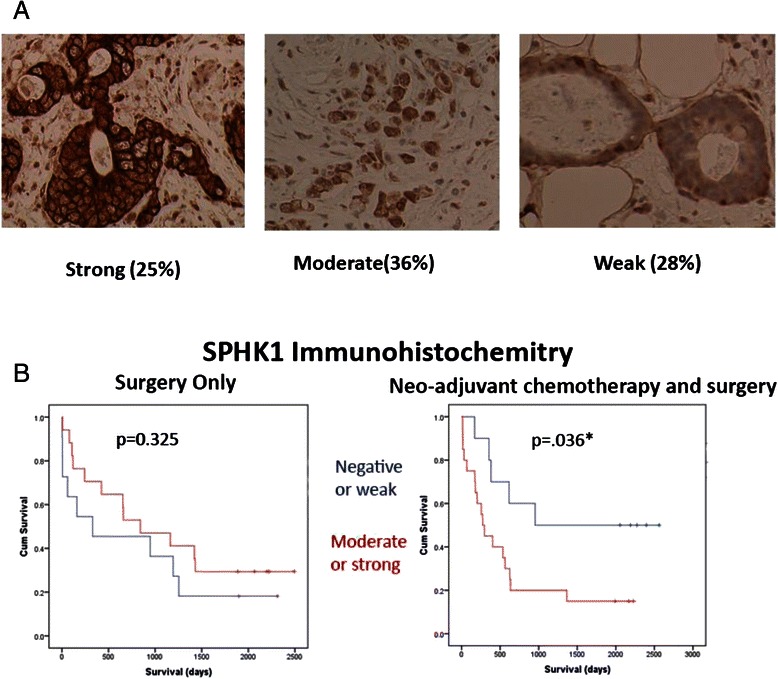
SPHK1 expression in gastroesophageal cancer patients. **a** SPHK1 Immunohistochemistry. Representative examples (x400) of strong, moderate and weak tumour SPHK1 staining and proportions of tumours in each category. **b** SPHK1 immunohistochemistry and overall survival of oesophago-gastric cancer patients treated with either surgery alone or with neoadjuvant chemotherapy prior to surgical resection, grouped negative or weak SPHK1 staining(blue line) versus moderate or strong SPHK1 staining (red line) (Kaplan- Meier survival curve, log rank test)

We examined the expression levels of SPHK1 protein by IHC in 67 gastroesophageal cancer patients (Table 1). We observed expression of SPHK1 protein in the cytosol in 60 (89 %) patients. There was no significant difference between the clinico-pathological characteristics of those patients that did and did not receive neo-adjuvant chemotherapy (Table 1). When SPHK1 staining was present, it was invariably present in virtually all tumour cells and we observed minimal variation in the proportion of tumour cells staining positive. The intensity of SPHK1 staining was variable between tumours, and was scored by 2 independent observers as negative, weak, moderate or strong (Fig. 5a). There was no association (*χ*^2^ test, *p* > 0.05) between SPHK1 staining as negative or weak versus moderate or strong and any of the clinco-pathological variables listed in table 1, in either entire cohort (*n* = 67) or the neo-adjuvant chemotherapy and surgery group (*n* = 36), nor the surgery alone group (*n* = 31). Higher expression of SPHK1 correlated with poor survival in patients treated with cisplatin based combination chemotherapy before surgery, but not those who received surgery alone without prior cisplatin based chemotherapy(surgery only patients median survival 841 days for SPHK1 moderate or strong versus 330 days for SPHk1 negative or weak, HR = 0.79, 95 % CI 0.65-1.4, *p* = 0.0325 and neo-adjuvant chemotherapy followed by surgery patients median survival 273 days for SPHK1 moderate or strong versus 954 days for SPHk1 negative or weak, HR = 1.67, 95 % CI 1.02-2.76, *p* = 0.036 Figure 5b). Only Tumour stage remained significant in a multivariate analysis with the input variables SPHK1 (negative or weak versus moderate or strong), surgical resection margins(positive versus negative), histology (squamous versus adenocarcinoma), site (oesophagus versus gastric) neo-adjuvant chemotherapy (yes versus no) and tumour stage (I or II versus III).

### Safingol reverses cisplatin resistance in a gastric adenocarcinoma cell line

**Fig. 6:**
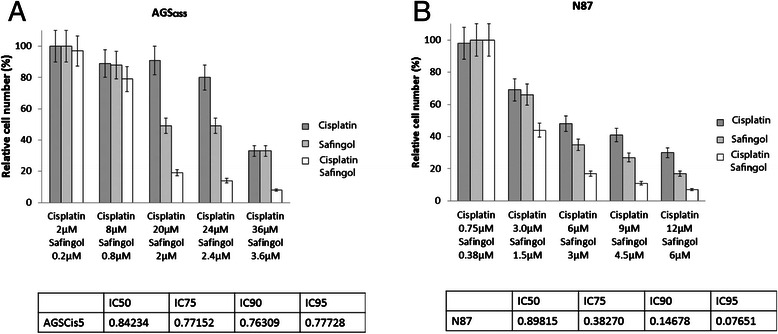
Synergistic effects of cisplatin and safingol in gastroesophageal cancer cell lines. **a** AGS_CIS5_ cisplatin resistant gastric cancer cell line and **b** N87 gastric cancer cell line. Cisplatin: Safingol ratio is constant in the combination experiments, and each data point has 6 replicates. Mean growth from three independent experiments shown, relative cell survival ((MTT OD value for cells treated as indicated /MTT OD value for untreated control)*100, ±SEM). Tables show combination index for cisplatin and safingol at different ICs for each cell line and show synergy between the treatments across different doses

We investigated the ability of safingol, an inhibitor of SPHK1, to reverse cisplatin resistance in gastric adenocarcinoma. The combination of safingol and cisplatin has been evaluated in a Phase I trial in solid tumours and is a safe well tolerated combination [38]. Safingol had cytotoxic activity as a single agent and also increased the cisplatin sensitivity of the highly cisplatin resistant cell line AGScis5, and also the gastric cancer cell line N87. In both cases cisplatin and safingol acted synergistically with the combination index suggesting strong synergy (Figs. 6a and b).

## Discussion

We approached the clinical need to identify predictive biomarkers for cytotoxic chemotherapy and new therapeutic targets in gastroesophageal cancer by using a hypothesis generating approach with global gene expression profiling of a panel of cell lines selected for resistance to clinically used cytotoxics. In order to enable rapid clinical translation, we prioritised further investigation of identified lead candidates for which agents already exist, as these would provide new strategies for combinations of targeted and cytotoxic therapies to overcome resistance and increase clinical effectiveness.

Sphingosine metabolism was identified as a lead candidate target following gene expression profiling and biological pathway mapping. In drug resistant gastroesophageal cancer cell lines we observed increased levels of sphingosine metabolite, S1P and increased cisplatin sensitivity in response to pharmacological inhibition of SPHK1, a kinase required for metabolism of sphingosine to S1P, as well as correlation between SPHK1 protein expression and cisplatin sensitivity in an independent gastric cancer cell line panel. Furthermore, SPHK1 protein expression was associated with worse survival in a cohort of patients with gastroesophageal cancer who received cytotoxic neo-adjuvant chemotherapy. Our data demonstrate an inverse relationship between the expression of SPHK1 (increased) and SGPL1 (decreased) in resistant cell lines and we propose this leads to increased cellular S1P and cytotoxic drug resistance.

S1P is a phospholipid with many functions, formed intra-cellularly through the phosphorylation of sphingosine by sphingosine kinases (2 isoforms SPHK1 and SPHK2) [39]. S1P is actively transported out of the cytosol to act via membrane S1P receptors, although receptor independent effects, including intracellular targets are also recognised [39]. Alternatively, irreversible cleavage of S1P by SGPL1 can occur in the cytosol. A role for SPHK1 and S1P in drug resistance in gastroesophageal cancer is consistent with many previous investigations, which have suggested a pathogenic role in several cancer types including gastric cancer [21], where SPHK1 overexpression is observed in tumour cells and associated with increased stage and poor survival [20]. In addition, investigations in cancer and non-cancer cells demonstrate that increased SPHK1 is associated with increased production of S1P in cells and S1P promotes cell proliferation, angiogenesis and inhibits cell death all of which could promote cell survival following cytotoxic drug insult and hence induce resistance [22–29, 37, 40, 41]. In addition SPHK1 activity and levels of S1P have been demonstrated to be involved in resistance to cytotoxic and targeted agents in a variety of cancer types, although not oesophageal or gastric adenocarcinoma drug resistance [30, 31, 34–36, 40, 42]. Here we provide the first evidence for the importance of sphingosine metabolism, and in particular SPHK1 and S1P, in resistance to cytotoxics in gastroesophageal cancer.

Accordingly S1P could lead to cytotoxic drug resistance in gastroesophegal cancer acting in an autocrine or paracrine manner via cell surface S1P receptors following transportation out of the cytosol. Alternatively S1P may mediate cytotoxic drug resistance acting intracellularly by counteracting apoptosis mediated by its pro-apoptotic precursor ceramide or interaction with known intracellular targets involved in cancer pathogenesis and cytotoxic drug resistance such as Histone deacetylase 1 (HDAC1) and Histone deacetylase 2 (HDAC 2) to which S1P directly binds and inhibits, and TNF Receptor-Associated Factor 2 (TRAF 2), or Protein Kinase C (PKC) [39]. Further investigation is required to determine which of these potentially pleomorphic mechanisms is important for cytotoxic drug resistance in gastroesophageal cancer, and the key mechanisms may be different for different drugs. Nevertheless our data demonstrates that S1P production controlled by SPHK1 and SGPL1 are key determinants of cytotoxic drug resistance and that decreasing S1P production in cancer cells could lead to increased cytotoxic sensitivity.

Rex et al. demonstrated no effect on the viability of cancer cell lines (cultured with and without serum starvation), nor on the growth of xenografts, with the use of highly specific SPHK1 and SPHK2 inhibitors [43]. However, the effect of SPHK1/2 inhibition under conditions of exposure to cytotoxic drugs was not investigated. Accordingly, it is not possible to rule out the importance of S1P and SPHK1 on tumour cell viability in specific circumstances not tested in their experiments- which would include exposure to cytotoxics. In addition it is possible that SPHK1 may have different effects in different types of cancer, and while Rex et al., provide data on a variety of cancer types, they have not provided data on gastroesophageal cancer cell lines. Our experiments with gastric adenocarcinoma cell lines suggest that safingol, a SPHK1 inhibitor, has cytotoxic effects as a single agent as well as acting synergistically with cisplatin.

Our finding of an inverse relationship between SPHK1 and SGPL1 expression in gastroesophageal cancer associated with increased levels of S1P and cytotoxic drug resistance is consistent with a recent report in prostate cancer, where a similar inverse relationship between expression of SPHK1 and SGPL1 was noted leading to increased production of S1P and an association with resistance to docetaxel [37]. In addition, increased S1P in glioblastoma multiforme tissues is associated with increased SPHK1 and decreased expression of S1P phosphatase (SGPP2) [22]. Together with our data, these observations suggest that increased SPHK1 and decreased SGPL1 or SGPP2 may be a relatively common pathogenic mechanism that could also be involved with therapy resistance in several cancer types.

Our data suggest that increased SPHK1 expression in gastroesophageal cancers has predictive impact indicating chemotherapy resistance in gastroesophageal cancer patients, consistent with our cell line findings, but not therapy independent prognostic value. A previous study by Li et al., demonstrated that high SPHK1 expression in resected gastric cancers was associated with worse survival [20]. Li et al., noted that the relationship between increased SPHK1 and survival was only significant for patients with higher stage (III and IV) disease who were often given adjuvant chemotherapy (chemotherapy details not provided) after surgery and there was no significant correlation between SPHK1 and survival for early stage (I and II) disease patients who were not given adjuvant chemotherapy after surgery. Given our findings reporting an effect of SPHK1 on resistance to chemotherapy, this interaction between SPHK1 and chemotherapy may explain the contrasting findings by Li and colleagues in early versus late stage disease. Pan et al., reported worse survival in esophageal squamous cell carcinoma patients treated with surgery alone (no neo-adjuvant chemotherapy) who had high SPHK1 protein expression [19]. Therefore, the impact of SPHK1 may vary according to histological sub-type of gastroesophageal cancer. Our investigation included 1 squamous cell carcinoma oesophagus cell line (OE21), but only a minority (18 %) of patients in our clinical cohort had squamous cell carcinomas. There was no observed association between SPHK1 IHC and histology and multivariate analysis did not suggest and differential effect of SPHK1 in squamous versus adenocarcinoma (although numbers were small in this analysis). Further prospective studies are required to determine the interaction between SPHK1 (SGPL1 and/or S1P) and histological subtype and their therapy independent prognostic versus predictive impact.

Here we have investigated the relationship between resistance and benefit from neo-adjuvant chemotherapy in gastroesophageal cancer patients. It would be valuable to investigate the relationship between SPHK1 expression and response to palliative chemotherapy to determine if there is any differential impact according to the stage of the disease. This would be useful in planning future clinical trials of therapies targeting SPHK1. The small numbers of patients in our surgical cohort who would have recurred and received palliative chemotherapy mean that such an analysis in our cohort would not be informative. In addition there may be a change in the SPHK1 expression from the primary tumour that was resected and the recurrent disease at a later date when palliative chemotherapy might be administered. Investigation in a large cohort of patients with advanced gastroeosphageal cancer that have received palliative chemotherapy would be worthwhile. Further investigation of the relationship between the ratio of SPHK1 and SGPL1 protein expression determined by immunohistochemistry or other methods, and the response/benefit from both neo-adjuvant and palliative chemotherapy in clinical cohorts of patients would also be valuable.

There are a number of SPHK1 and S1P inhibitors in clinical development as anti-cancer agents that confer increased sensitivity to cytotoxic drugs, targeted agents or radiotherapy and/or have single agent activity in preclinical cancer models [30, 31, 38, 44–49] (Table 2). Therefore our findings in gastroesophageal cancer could be readily translatable to the clinic. SPHK1 may be a useful biomarker to identify patients who are likely to be resistant to cytotoxic chemotherapy and who would benefit from the addition of a SPHK1 or S1P inhibitor to a cytotoxic chemotherapy regimen. Alternatively, SPHK1 and S1P inhibitors may be useful as part of second line therapies in patients who have clinical resistance to cytotoxic chemotherapy, a situation where current therapies have limited efficacy. We investigated this *in vitro* by examining the effect of safingol on cisplatin resistance in gastric cancer. We chose safingol (L-theo dihydrospingosine) rather than a more specific SPHK1 inhibitor or SPHK1 knock down by siRNA mediated knock down of SPHK1, since it is the most developed SPHK1/S1P inhibitor as an anti-cancer agent and in particular safingol has demonstrated safety in combination with cisplatin in a phase I clinical trial and lead to decreased serum S1P in treated patients [38]. Accordingly using safingol would provide useful data to facilitate more rapid translation to a clinical therapy and similar to the phase I trial, we investigated the combination of cisplatin safingol in gastrooesophegal cell lines. Safingol is a potent competitive inhibitor of SPHK1 with a Ki <0.4 μM, it is a substrate for but not inhibitor of SPHK2, and it is also known to inhibit PKC but the Ki is considerably higher at 33–40 μM [50–53]. In addition safingol treatment of colon cancer cells leads to decreased S1P and increased sphingosine [46]. In our evaluation of safingol in the cisplatin resistant cell line AGS_CIS4_ and the gastric cancer cell line N87 we used safingol at concentrations ranging from 0.375–12 μM, which is well within the readily achievable serum plasma concentrations of safingol in cancer patients and at which significant decreases in serum S1P are reported [38]. As well as synergy with cisplatin our experiments demonstrated cytotoxic activity of safingol, which together with the safety data from the clinical phase I trial with concomitant cisplatin administration suggest that this would be a feasible and appropriate combination to investigate in early phase trials in gastroesophageal cancer patients. Further pre-clincial experiments with specific SPHK1 inhibitors and siRNA mediated knockdown of SHK1 would allow additional exploration of the potential of SPHK1 as a target and the usefulness of combining with other cytotoxic and targeted drugs.

**Table 2:** SPHK1 and S1P inhibitors in clinical development as anti-cancer therapies

Agent	Mechanism	Stage of Clinical Development	References
Safingol	SPHK1 inhibitor	Demonstrated safety in combination with cisplatin in Phase I trial in solid tumours	[43, 46, 47]
Fingolimod	S1P receptor antagonist	Licenced for use in multiple sclerosis. No data on clinical use in cancer	[29, 30, 44]
(FTY720)			
Sonepcizumab	Humanised S1P neutralising monoclonal antibody	Demonstrated safety in Phase I trial in solid tumours	[48, 49]
(LT1009)			

## Conclusion

In conclusion, we have demonstrated for the first time in gastroesophageal cancer that increased SPHK1 occurs in combination with decreased SGPL1 and increased S1P, and cytotoxic drug resistance, and that a SPHK1 inhibitor safingol can reverse cisplatin resistance. Given the demonstrated clinical safety and tolerability of safingol in combination with cisplatin, as well as the availability of several other SPHK1 and S1P inhibitors in stage clinical development, we suggest that combining such agents with cytotoxic chemotherapy or investigating their activity in patients who have developed secondary resistance to chemotherapy is a promising clinical strategy and that a high tumour SPHK1:SGPL1 ratio could provide a predictive biomarker to select patients for this therapeutic approach.

## Additional file

Additional file 1: **Additional information 1.** Additional information on generation of drug resistant cell lines. **Additional information 2.** Details of sample preparation and gene expression profiling. **Additional information 3.** Gene expression Data. **Additional information 4.** PCR primer sequences. **Additional information 5.** Additional details of analysis and quantification of sphingosine-1-phosphatefrom cell lines using high performance liquid chromatography-tandem mass spectrometry (HPLC-MS/MS). **Additional information 6.** Additional details of chemotherapy treatment of patients. **Additional information 7.** Pathways identified in Gene enrichment analysis (DAVID v6.7) of resistant cell lines. (PDF 725 kb)

## Abbreviations

EGFR: Epidermal Growth Factor Receptor
HDAC1: Histone deacetylase 1
HDAC 2: Histone deacetylase 2
IC50: Half maximal inhibitory concentration
mTOR: Mammalian Target of Rapamycin
PKC: Protein Kinase C
qRT-PCR: Quantitative real-time polymerase chain reaction
SPHK1: Sphingosine kinase 1
S1P: Sphingosine-1-phosphate
TRAF 2: TNF Receptor-Associated Factor 2
VEGF: Vascular Endothelial Growth Factor
