# Supplementary material for: Regulation of cellular sphingosine-1-phosphate by sphingosine kinase 1 and sphingosine-1-phopshate lyase determines chemotherapy resistance in gastroesophageal cancer
Source: BMC Cancer. 2015 Oct 22;15:762. doi: 10.1186/s12885-015-1718-7 (PMC4618539; doi:10.1186/s12885-015-1718-7)
Supplement: Additional file 1: — Additional information 1. Additional information on generation of drug resistant cell lines. Additional information 2. Details of sample preparation and gene expression profiling. Additional information 3. Gene expression Data. Additional information 4. PCR primer sequences. Additional information 5. Additional details of analysis and quantification of sphingosine-1-phosphatefrom cell lines using high performance liquid chromatography-tandem mass spectrometry (HPLC-MS/MS). Additional information 6. Additional details of chemotherapy treatment of patients. Additional information 7. Pathways identified in Gene enrichment analysis (DAVID v6.7) of resistant cell lines. (PDF 725 kb) [file 12885_2015_1718_MOESM1_ESM.pdf]

Supplementary Information-1

Additional information on generation of drug resistant cell lines

Oesophagogastric cancer cell lines were grown in incrementally increasing concentrations of cisplatin, oxaliplatin and docetaxel(μM range) over 24 months. Bars represent measurements of cell viability that were taken after every 4 passages. X-axis indicates various cell lines, Y-axis resistance index (RI) defined as a fold change of IC50 values between each pair of resistant versus parental line. Error bars were calculated for 3 independent replicates and defined as SEM.

Figure S1A-Cisplatin resistant cell lines

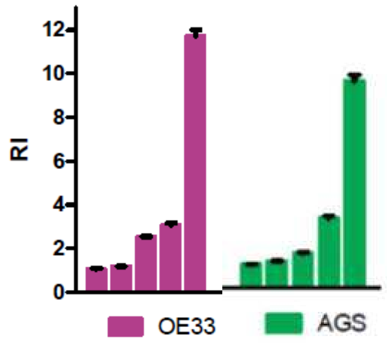

Figure S1B-Oxaliplatin resistant cell lines

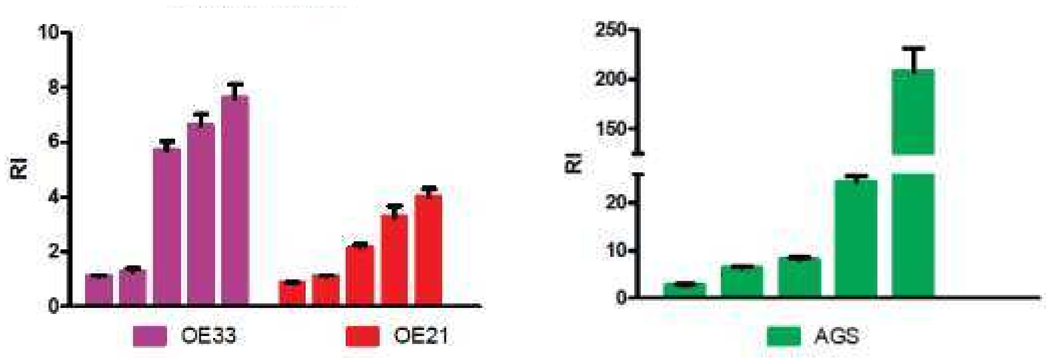

Figure S1C-Docetaxel resistant cell lines

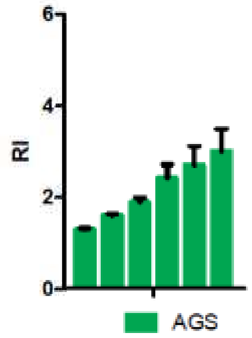

Supplementary Information-2

Details of sample prepapration and gene expression profiling

RNA Extraction

Cells were grown in T75 flasks to 80 – 90 % confluency and washed twice in PBS. 1 ml of TRIzol (Invitrogen, Paisley, UK) reagent was added per flask and then lysates were transferred into 1.5 ml eppendorf tube and passed through a 20 gauge needle (0.9 mm diameter) at least 5-6 times. Samples were centrifuged at 1200g for 10 minutes at 4 °C and supernatants were collected and transferred into fresh tubes and incubated for 5 minutes at room temperature (RT). 200 µl of chloroform (Sigma Aldrich) was added and tubes were inverted several times and incubated for 3 minutes at RT. Tubes were centrifuged at 12000 g for 15 minutes at 4 ^{o}C. Supernatants were collected and transferred into new eppendorf tubes. 500 µl of 100 % isopropanol was added, mixed and incubated for 10 minutes at RT. Samples were centrifuged at 12000 g for 10 minutes at 4 °C. Supernatants were removed and pellets washed with 1ml of 75 % ethanol, vortex and centrifuged at 9500 rpm for 5 minutes at 4 °C. Remaining supernatants were removed, pellets briefly air-dried and resuspended in 30 µl of RNase free water. RNA was quantified spectrophotometrically (Nanodrop 1000 Spectrophotometer, Thermo Scientific, Loughbourough, UK). Both A260/280 and A260/230 ratios were determined and all samples were in the range 1.9- 2.3. Furthermore samples were purifed on the Mini columns (Qiagen). Quality was assessed by electrophoresis on Tapestation (Lab901 Limited, Peqlab, UK) and QC was determined by SDV (Screen Tape degradation value) that represents RNA integrity. Values 0-5 represent high quality RNA, 5-14 - partially degraded RNA and ≥ 15 degraded RNA. All RNA samples had SDV values between 0.3 and 2.6. All analyses used 3 independent replicates per cell line from three different passages.

Sample preparation for Gene expression profiling and hybridisation

500 ng of total RNA was reverse transcribed into cDNA and further amplified in vitro into cRNA using Ambion WT Expression kit (Austin, TX, USA). The quality of cRNA was determined by electrophoresis (Tapestation, Lab901 Limited, Peqlab, UK). The A260/280 and A260/230 ratios, concentration and yield were determined on a spectrophotometer (Nanodrop 10000 spectrophotometer, Thermo Scientific, Loughborough, UK). cRNA (10µg) was reverse transcribed into cDNA (Ambion WT Expression Kit, Ambion) and its quantity and quality was determined as above. Subsequently, cDNA samples (5.5µg) were fragmented and biotin labelled (WT terminal labelling and controls kit, Affymetrix, Santa Clara, CA), and 5.5 µg hybridised to Human Exon 1.0 ST GeneChip microarrays (Affymetrix, Santa Clara, CA) at 45 °C for 17 hours at 45 rpm in a hybridization oven.

Supplementary Information-3

Gene expression Data

Quality assessment(QA) of Gene expression data

QA data for the profiling of drug resistant and parental cell lines is provided in table S4.1 and figure S4.1 below.

Core probe sets on the Human Exon 1.0 ST array were processed using a modified robust multiarray analysis (RMA16) algorithm (Affymetrix, Santa Clara, CA) that employs a non-linear per chip background correction with addtion of 16 to the expression values to attain variance stabilisation of low level signals, quantile normalisation and summarisation of multiple probe sets per transcript using median polishing of log2 transformed data. Data were transformed to the median of all samples. QA was performed by examining signal intensity (PM_mean), background signal detection (Bgrd_mean), detection of outliers followed by analysis of hybridization and labelling controls. Further determination of outlier samples was performed by analysis of probe set summarization metrics such as Pos_vs_neg_auc and All_Probe_Set_RLE_Mean.

Quality control of hybridization and labelling was determined by analysis of bacterial (bac_spike) and polyadenylated (polyA_spike) controls and polyadenylated RNA spikes such as Lys, Phe, Thr and Dap were analyzed independently.

Table S4.1 Quality assessment measures from Affymetrix 1.0ST Exon arrays for all cell line samples

Signal detection	Range	Detected across all samples
**PM_mean**	200-400	Within the range
**Bgrd_mean**	200-550	Within the range
**Summarization metrics -outliers**		
**Pos_vs_neg_auc:**	0.8 -0.9	Within the range
**All_Probe_Set-RLE_mean**	Close to the median	-2.67 - 1.41
**Hybridization QC**		
**PolyA**	4-5	Within the range
**Lys, Phe, Thr,Dap**	7-8	Within the range

Figure S4.1 Box plots represent the relative log expression for all the probe sets analyzed. The mean absolute RLE is proportional to the width of the box plots, or the inter-quartile range of RLE values and whiskers are 1.5x IQR.

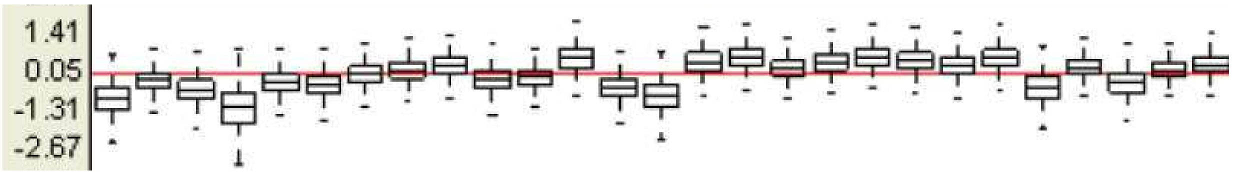

Analysis of gene expression data

Gene expression data analysis was performed in GeneSpring GX v 11.5 using RMA16 normalization, log transformation and baseline to median of all samples. Core gene sets were analysed andentities with normalised expression levels between the 20th and 100th percentiles in at least 1 sample were included with 16939 out of 17881 genes meeting this filter. Unpaired t-test, with Benjamini and Hochberg MTC corrected $p \leq 0.05$ was performed to identify discriminatory gene profiles of each pair of drug resistant versus parental lines.

Pathway analysis

Gene set enrichment analysis using gene ontologies and mapping of gene sets of interest onto biological pathways was performed using DAVID v 6.7 (Function Annotation Bioinformatics Microarray Analysis), Bioinformatic Resources, NIAID, NIH Significantly enriched GO terms, functional networks or pathways were determined using gene set enrichment analysis (EASE score, DAVID v 6.7) and genes were visualised on BioCarta and KEGG pathways. Data was pre-processed in DAVID bioinformatics database to provide the link between probe IDs and Entrez Gene Symbols. Probe sets with multiple Entrez IDs and those which could not be found in Ensembl/Entrez ID were excluded from further analysis.

Supplementary information -4

PCR primer sequences

Primer Name	Length	Sequence (5'-3')
SPHK1 Forward	20	ATCCAGAAGCCCCTGTGTAG
SPHK1 Reverse	19	TGGTGACCTGCTCATAGCC
SGPL1 Forward	19	GGGTCCCATTGACGAAGAT
SGPL1 Reverse	20	TGGCAGTGTTCCTGGAGATA
GAPDH Forward	19	AGCCACATCGCTCAGACAC
GAPDH Reverse	19	GCCCAATACGACCAAATCC

Suplementary Information-5

Additional details of analysis and quantification of sphingosine-1-phosphate from cell lines using high performance liquid chromatography-tandem mass spectrometry (HPLC-MS/MS)

Equipment used for Tandem mass spectrometry (MS/MS): Thermo Fisher TSQ Quantum (Thermo Fisher Scientific); HPLC analysis: Agilent-1100 (Agilent Technologies); column: Spectra C8SR (Peeke Scientific, Redwood City, CA) 150 x 3.0 mm, 3-µm particle size.

For cell pellet preparation and lipid extraction for analysis, cells were washed 3 times in ice cold phosphate buffered saline (PBS), pH 7.4, scraped on ice in 10 ml of ice cold PBS using a cell scraper (Corning,UK) and centrifuged at 500 x g for 5 minutes at 4°C. PBS was removed and cell pellets were fortified with 50µl (1nmole/ml) internal standard solution.

Lipids were extracted from cell pellets by adding 2ml of iso-propanol-water-ethyl acetate (30:10:60 vol), sonication for 30 sec, vortexing and centrifugation for 6 minutes at 3000 x g. Organic upper phase was transferred into a new tube and the remaining sample was re-extracted as previous. The new organic phase was combined with the previous supernatant and 1ml of lipid extract was dried down to determine phosphorus concentration (Pi). The remaining organic phase was evaporated under Nitrogen to dryness. Dry residue were reconstituted in 150 µl of the mobile phase "B" containing 2mM ammonium formate solution in methanol containing 0.2 % formic acid, then centrifuged for 5 minutes at 3000 x g. Samples (200 µl) were transformed to an auto–sampler HPLC vial and 10 µl were injected into the HPLC system. The phosphate content was measured with a standard curve and a colorimetric assay of ashed phosphate.

Supplementary Information-6

Additional details of chemotherapy treatment of patients

Neoadjuvant chemotherapy was with Epirubicn, Cisplatin , and capecitabine (epirubicin 50mg/m2 intravenous day 1, cisplatin 60mg/m2 intravenous day1 and capecitabine 1250mg/m2/day in 2 divided doses orally day 1-21, with a 21 day cycle) .Radiological response was assessed by CT chest and abdomen pre-therapy and after 3 (neoadjuvant) or 4 (palliative) cycles of chemotherapy according to RECIST criteria v1.1. If the absence of progressive disease on CT scan, patients receiving neoadjuvant chemotherapy proceeded to surgical resection after 3 cycles and also 3 cycles of post-operative/adjuvant chemotherapy with the same regimen if the treating oncologist considered them fit enough to begin this treatment within 12 weeks of the resection .

Supplementary Information-7: Pathways identified in Gene enrichment analysis (DAVID v6.7) of resistant cell lines

The folowing tables show the pathways and genes were identified as over-represented from the lists of genes with significantly different expression (student's t-test p<0.05) between resistant daughter cell line and parental wild type cell line using DAVID v6.7, p<0.05 with Benjamini and Hochberg correction used for multiple testing.

AGSCis5 verus AGS

AGSCIS5 DAVID		
**Term**	**Genes**	**p values**
Lysosome	ARSB, GM2A, HEXA, LGMN, AP3S2, CTSA, GLB1, CD68, TPP1, LIPA, PSAP, GUSB, ATP6V1H, CD63, FUCA1, GNS, LAMP1, LAMP3, GLA, IGF2R, GAA, CTSD, CTSB, CTNS, GGA2, CTSH	7.237E-06
Base excision repair	POLD4, POLE3, UNG, LIG1, POLE, LIG3, PARP3, XRCC1, APEX1, FEN1	0.001718
DNA replication	POLD4, MCM7, POLE3, RFC2, LIG1, POLE, MCM2, MCM4, FEN1, MCM5	0.0021262
p53 signalling pathway	STEAP3, PMAIP1, SFN, CCNG2, GTSE1, CCNE1, TP53I3, CDKN1A, TNFRSF10B, CD82, SERPINE1, DDB2, THBS1, GADD45A	0.0033654
Pyrimidine metabolism	POLR3H, POLE, POLR1A, CAD, POLR3GL, TK2, POLD4, TYMS, POLE3, ENTPD5, CDA, TXNRD1, UCK2, DPYD, DUT	0.0238725
Proteasome	PSMC5, PSME1, PSMC4, PSME2, PSMD11, PSMD2, PSMD8, PSMB8, PSMB9	0.0374423
ECM-receptor interaction	HSPG2, DAG1, ITGB4, ITGA3, SDC4, LAMB3, LAMB2, LAMA3, CD44, ITGA5, LAMC2, THBS1, SPP1	0.0430795
Fatty acid metabolism	ACADVL, ACAA2, CPT2, ACSL1, ALDH1B1, ACAT2, PECI, ACSL5	0.0438907
Glutathione metabolism	GSTM1, GPX2, ODC1, GSTM4, SRM, GSTK1, GPX3, IDH2, MGST2	0.0497812
Cell cycle	E2F4, CDC14B, CREBBP, SMAD3, CDC20, MCM2, SFN, CDC25C, MCM4, ORC1L, CDC25A, MCM5, CCNE1, CDKN1A, MCM7, PLK1, GADD45A	0.0515377
Galactose metabolism	GLA, PGM1, GAA, HK1, PFKM, GLB1	0.0525463
Systemic lupus erythematosus	HIST1H2AB, HIST1H2AC, HIST1H4L, HIST1H2AE, HLA-DMA, HIST2H2AB, HIST1H2BM, HIST1H2BN, HIST1H4B, HIST1H4E, H2AFX, HIST1H4C, HIST1H4D, HIST1H4H, HIST1H3J, HIST1H2BB, HIST1H2BC, HIST1H2BF, HIST1H2BG, TRIM21, HIST2H2BF, HIST1H3A, HIST1H3B, HIST1H2AH, HIST1H3C, HIST1H2AK, HIST1H3D, HIST1H2AM, HIST1H3F, HIST1H3G, HIST1H3H, HIST1H3I	0.0591349
Antigen processing and presentation	HSP90AB1, PSME1, PSME2, TAP2, LGMN, TAP1, HLA-B, CTSB, HLA-E, HLA-DMA, TAPBP, B2M	0.0641706
Glycosaminoglycan degradation	ARSB, GNS, HEXA, GUSB, GLB1	0.0799584
Sphingolipid metabolism	SGPL1, GLA, SGPP1, SGPP2, PPAP2A, SMPD4, GLB1	0.0906448

AGSOX8 versus AGS

AGSOX8 DAVID		
**Term**	**Genes**	**p values**
Valine, leucine and isoleucine degradation	ALDH6A1, ACAD8, HADH, HADHA, AUH	0.0333705
Pathways in cancer	BMP4, HSP90AB1, RALBP1, GRB2, CYCS, FGF20, TCF7L2, STAT3, TGFB2, RAD51, IGF1R, CBLB, CDKN1B, LAMC2, RUNX1	0.0724271
Fatty acid metabolism	ACSL1, HADH, PECI, HADHA	0.0997143

AGSDOC6 verus AGS

AGSDOC6 DAVID		
**Term**	**Genes**	**p values**
p53 signaling pathway	BID, STEAP3, ZMAT3, CYCS, RRM2B, PMAIP1, CDK4, CCNG2, GTSE1, CCNE1, CCNB3, CCND1, TNFRSF10B, CASP9, SERPINB5, SERPINE1, MDM2, APAF1, FAS, PERP, THBS1, GADD45A	2.296E-05
Pyrimidine metabolism	POLR2F, POLR2L, ZNRD1, CAD, RRM2B, TK1, POLR2A, POLE4, POLE3, ENTPD5, CDA, UCK2, NT5C, NUDT2, POLR1D, POLE, POLR1A, POLR3GL, POLR1C, NME6, NME4, POLD1, POLD2, UCKL1, TXNRD1, DUT	8.061E-05
Steroid biosynthesis	TM7SF2, SOAT1, EBP, LIPA, DHCR7, LSS, DHCR24, FDFT1	0.0021861
Apoptosis	BID, IRAK1, TRAF2, CFLAR, DFFA, RELA, DFFB, CYCS, ENDOD1, BCL2L1, TNFRSF10A, AKT1, CASP10, PRKAR2A, TNFRSF10B, MYD88, CASP9, PPP3CC, APAF1, FAS, AKT2	0.0025269
Pathways in cancer	FGF6, HSP90AB1, E2F2, FGF18, WNT3A, TGFB1, MMP1, AKT1, ACVR1B, CCNE1, CASP9, CDKN2B, SLC2A1, RHOA, RALB, FAS, AKT2, PRKCA, EGFR, CTBP1, RALBP1, RXRB, RELA, CYCS, FGF20, CDK4, STK4, CCND1, PDGFRA, MDM2, LAMC1, PIAS1, BID, FGFR2, TRAF2, GRB2, ERBB2, EGLN2, BCL2L1, IGF1R, AXIN2, RUNX1, BMP4, DVL3, EPAS1, MET, CREBBP, ITGA2, BIRC5, ITGA3, FZD5, FZD7, CDKN1B, ETS1, ABL1	0.0071809
DNA replication	POLE4, MCM7, POLE3, RFC2, POLD1, LIG1, POLD2, POLE, MCM2, RNASEH2A, MCM5	0.0075846
Small cell lung cancer	E2F2, TRAF2, RXRB, RELA, CYCS, ITGA2, ITGA3, BCL2L1, CDK4, AKT1, CCNE1, CCND1, CDKN1B, CASP9, CDKN2B, PIAS1, APAF1, LAMC1, AKT2	0.0088063
Inositol phosphate metabolism	INPP1, PIK3C2B, SYNJ1, PI4KA, ITPKB, PI4KB, PLCB3, PLCB4, PIK3C3, PIP4K2A, ITPK1, IPMK, INPP5A, PIP4K2B	0.0092146
Phosphatidylinositol signaling system	PRKCA, INPP1, PIK3C2B, SYNJ1, PI4KA, ITPKB, PI4KB, ITPR3, PLCB3, PLCB4, PIK3C3, CALM3, INPP5D, PIP4K2A, ITPK1, CALM2, INPP5A, PIP4K2B	0.0120426
Spliceosome	DHX8, PRPF31, CCDC12, SF3B14, U2AF2, LSM7, HSPA1A, SF3A1, SF3B4, SART1, SF3B3, PRPF6, SFRS4, DDX23, DHX38, PRPF8, SNRPB, PQBP1, DHX16, SNRPA, ACIN1, PHF5A, PUF60, BAT1	0.0230871
Glutathione metabolism	GPX2, GSTM2, ODC1, GSR, GSTM3, GSTM4, SRM, GPX5, PGD, GSTO2, RRM2B, GCLM	0.0303956

AGSDOC6 verus AGS (continued)

AGSDOC6 DAVID		
**Term**	**Genes**	**p values**
Prostate cancer	EGFR, FGFR2, HSP90AB1, E2F2, GRB2, ERBB2, RELA, CREBBP, AKT1, CCNE1, IGF1R, CCND1, CDKN1B, CASP9, INS, PDGFRA, MDM2, AKT2	0.0316127
Sphingolipid metabolism	SGPL1, SPTLC2, SGPP1, KDSR, B4GALT6, PPAP2A, SMPD4, SMPD2, ASAH2, DEGS1	0.0362435
Colorectal cancer	EGFR, DVL3, GRB2, CYCS, MET, BIRC5, FZD5, TGFB1, FZD7, AKT1, ACVR1B, IGF1R, CCND1, CASP9, PDGFRA, AXIN2, AKT2	0.0372152
Purine metabolism	POLR2F, GDA, POLR2L, ADCY8, ZNRD1, RRM2B, POLR2A, POLE4, PDE6D, POLE3, ATIC, ENTPD5, IMPDH2, NT5C, NUDT2, POLR1D, POLE, POLR1A, POLR1C, POLR3GL, NPR2, PDE4D, NME6, NME4, PKM2, POLD1, POLD2	0.0374239
RNA polymerase	POLR2F, POLR2L, POLR1D, POLR1A, POLR1C, POLR3GL, ZNRD1, POLR2A	0.0416694
Parkinson's disease	ND1, NDUFA3, SNCAIP, UQCRC1, COX8C, ND5, ATP5B, CYCS, ND3, CYC1, NDUFC1, UQCRFS1, ATP5G3, NDUFV3, NDUFS6, ND4L, CASP9, UBA1, COX6B1, APAF1, SLC18A1, ND6, ATP5J	0.0472622
Base excision repair	POLE4, POLE3, UNG, POLD1, LIG1, POLD2, POLE, XRCC1, OGG1	0.0494558
Non-small cell lung cancer	PRKCA, EGFR, AKT1, E2F2, CCND1, CASP9, GRB2, RXRB, ERBB2, CDK4, STK4, AKT2	0.0504121
Chronic myeloid leukemia	E2F2, CTBP1, GRB2, RELA, BCL2L1, CDK4, TGFB1, AKT1, ACVR1B, CCND1, CDKN1B, MDM2, ABL1, RUNX1, AKT2	0.0569907
Cell cycle	E2F2, RBL2, CREBBP, PKMYT1, CDC20, MCM2, CDK4, CDC25C, MCM5, TGFB1, CDC25A, CCNE1, CCND1, CCNB3, MCM7, CDKN1B, CDKN2B, PLK1, TFDP2, MDM2, ABL1, GADD45A	0.0634736
Pancreatic cancer	EGFR, E2F2, RALBP1, ERBB2, RELA, BCL2L1, CDK4, TGFB1, AKT1, ACVR1B, CCND1, CASP9, RALB, AKT2	0.080851
Adipocytokine signaling pathway	TRAF2, RXRB, LEPR, RELA, NFKBIB, PRKAG2, PRKAB2, IRS1, AKT1, SLC2A1, JAK2, AKT2, ACSL5	0.0955582
Lysosome	LIPA, CLTB, LAPTM4A, GM2A, PSAP, HEXA, LGMN, CTSA, ACP2, AP4M1, AP4S1, FUCA1, GNS, LAMP1, CD68, TPP1, IGF2R, ATP6V0A1, CTSH, GGA3	0.0976249

OX33CIS4 versus OE33

OE33CIS4 DAVID		
**Term**	**Genes**	**p values**
Lysosome	ARSB, SGSH, CLTB, LGMN, ATP6AP1, HEXA, AP3S2, AP3S1, ABCA2, CLTC, ATP6V0B, GLB1, AP1S3, AP1S1, CD68, AP1S2, AP3M2, AP3M1, GALC, ATP6V0D1, GBA, CLN3, CTSZ, LIPA, AP4M1, NPC1, GAA, CTSD, CTNS, GGA2, CLTCL1, CTSH, CLN5, GGA3	9.012E-05
Ribosome	RPL18, RPL15, RPL35, RPL36, RPL10L, RPL32, RPS29, RPL6, RPSAP58, RPLP1, RPL26L1, FAU, RPL11, RPL4, RPS24, RPL35A, RPSAP15, RPL27, RPL24, RPS8, RPS7, RPL29, RPS19, RPL18A, RPS15, RPS11, UBA52	0.0004637
Ubiquitin mediated proteolysis	UBE2Z, XIAP, UBE3B, UBE3A, UBA6, CDC34, ANAPC11, UBE3C, CUL3, CUL5, WWP1, ITCH, TRAF6, TRIP12, ANAPC2, VHL, FBXO2, BIRC6, UBE2L6, CDC23, MID1, CDC26, UBE2L3, CDC27, BRCA1, TRIM37, UBE2O, PIAS4, UBR5, SIAH1, CUL4B, SMURF1, UBE2E2, UBE2E1	0.0019726
Adherens junction	PTPRJ, PARD3, PTPRF, BAIAP2, NLK, ERBB2, LEF1, CTNND1, SMAD2, ACP1, TCF7L2, VCL, CSNK2A2, PVRL4, CDC42, MAPK1, ACVR1B, CSNK2A1, EP300, RHOA, YES1, MLLT4	0.0026415
Neurotrophin signaling pathway	GRB2, IRAK4, CDC42, MAP3K5, KRAS, MAP3K3, BCL2, RHOA, TRAF6, PIK3R3, FRS2, RAPGEF1, ARHGDIA, PDK1, IRAK2, TP53, RAF1, PRKCD, YWHAE, MAPK1, RPS6KA3, RPS6KA1, PLCG1, MAPK13, MAPK14, CALM3, RIPK2, MAPK7, IKBKB, CRK	0.0056639
Proteasome	PSMB5, PSMB4, PSMC6, PSMB7, PSMC5, PSMB6, PSMA6, PSMD12, PSME2, PSMA4, PSMB2, PSMD2, PSME4, PSMD6	0.0145108
Epithelial cell signaling in Helicobacter pylori infection	GIT1, ATP6V0E1, ADAM10, LYN, ATP6AP1, ATP6V1G1, ATP6V1B2, ATP6V0B, ATP6V1F, ATP6V1A, CDC42, PLCG1, MAPK13, MAPK14, ATP6V1E1, HBEGF, IKBKB, ATP6V0D1	0.0160035
p53 signaling pathway	STEAP3, TP53, CDK6, RRM2B, SFN, CCNG1, PTEN, ATM, CCNE1, CCND1, PPM1D, CDKN1A, CCNB2, SERPINB5, SHISA5, SIAH1, FAS, THBS1	0.0160035
Colorectal cancer	DVL2, MSH6, GRB2, TP53, LEF1, RAF1, SMAD2, FZD3, BIRC5, FZD2, FZD5, TCF7L2, RALGDS, FZD6, MAPK1, ACVR1B, CCND1, KRAS, BCL2, PIK3R3, AXIN1	0.0162303
Tight junction	PRKCZ, PARD3, CLDN9, ZAK, GNAI1, CLDN3, CASK, AMOTL1, PTEN, LLGL2, CSNK2A2, CDC42, KRAS, CSNK2A1, RHOA, EXOC4, YES1, MLLT4, PPP2R2D, PRKCH, PRKCG, MYH9, CSDA, PRKCE, PRKCD, PRKCQ, RAB13, TJP3, PPP2R2A, SPTAN1	0.0168732
Amino sugar and nucleotide sugar metabolism	UAP1, CYB5R1, GNPDA1, GNE, CMAS, HEXA, HK1, UXS1, GALK2, PGM2, MPI, GFPT1, GFPT2	0.0206507
Cell cycle	CDC14B, DBF4, SFN, ANAPC11, CCNE1, ORC2L, MCM7, ORC4L, BUB1, BUB3, STAG2, ANAPC2, TP53, CDC23, CDK6, SMAD2, CDC26, CDC27, YWHAE, ATM, SMC3, WEE1, MCM6, CDKN1A, CCND1, CCNB2, EP300, HDAC1	0.0211571
Vibrio cholerae infection	ATP6V0E1, SLC12A2, ATP6AP1, PRKCG, ATP6V1G1, ATP6V1B2, PRKX, ATP6V0B, ATP6V1F, ATP6V1A, SEC61B, PLCG1, ATP6V1E1, ATP6V0D1, SEC61A2	0.0273681
Prostate cancer	FGFR2, CREB3, GRB2, ERBB2, TP53, LEF1, RAF1, TCF7L2, PTEN, CCNE1, MAPK1, CDKN1A, CCND1, PDPK1, KRAS, EP300, BCL2, TGFA, MTOR, PIK3R3, IKBKB	0.0295084
Fc gamma R-mediated	LYN, LIMK1, SPHK1, ASAP2, ASAP1, RAF1, PRKCG, ARPC5, PRKCE, PRKCD, TTLL3, ARPC1A, CDC42, MAPK1,	0.0309516

OX33CIS4 versus OE33 (continued)

OE33CIS4 DAVID		
Term	Genes	p values
phagocytosis	ARPC1B, PLCG1, ARPC3, ARPC2, CFL2, PIK3R3, CRK, SYK	
Endometrial cancer	GRB2, ERBB2, TP53, RAF1, LEF1, PTEN, TCF7L2, MAPK1, CCND1, PDPK1, KRAS, ILK, PIK3R3, AXIN1	0.0328193
Endocytosis	FGFR2, PRKCZ, PARD3, CLTB, CHMP4C, TSG101, CHMP4B, CHMP6, ASAP2, ASAP1, ZFYVE20, CLTC, CDC42, ACVR1B, AP2B1, WWP1, SNF8, GIT2, ITCH, AGAP1, TRAF6, VPS36, GIT1, STAMBP, RUFY1, PSD4, RAB11FIP4, AP2A2, ARRB2, RABEP1, VPS24, HGS, SMURF1, CLTCL1, ARAP2, RAB11FIP1, VPS25	0.0371566
Progesterone-mediated oocyte maturation	ANAPC2, ADCY7, GNAI1, ADCY6, CDC23, RAF1, ANAPC11, CDC26, PPP1CB, CDC27, PRKX, MAPK1, RPS6KA3, CCNB2, KRAS, RPS6KA1, MAPK13, MAPK14, BUB1, PIK3R3	0.0390638
Thyroid cancer	MAPK1, CCND1, KRAS, NCOA4, PAX8, TP53, TFG, LEF1, TCF7L2	0.0508863
Insulin signaling pathway	PRKCZ, EXOC7, GRB2, PHKB, PHKA1, HK1, RHOQ, PRKX, PDPK1, PPP1R3B, KRAS, INPP5K, PRKAA2, TRIP10, PIK3R3, RAPGEF1, PTPRF, PRKAB1, RAF1, PPP1CB, RPTOR, MAPK1, PYGL, PRKAR1A, CALM3, MTOR, IKBKB, CRK	0.0513924
N-Glycan biosynthesis	MGAT4B, MAN2A1, MGAT4A, RFT1, TUSC3, ALG2, DAD1, RPN1, MOGS, MAN1A1, ALG8, DDOST	0.0630312
Pathways in cancer	PPARD, GLI2, PTEN, CDC42, ACVR1B, CCNE1, PAX8, RHOA, TGFA, FAS, FGF3, TP53, LEF1, PRKCG, CDK6, DAPK2, MAPK1, CCND1, EP300, PIAS4, NCOA4, VEGFA, WNT9A, FGFR2, XIAP, GRB2, ERBB2, TFG, EGLN2, TCF7L2, LAMB3, KRAS, BCL2, LAMB1, PIK3R3, TRAF6, AXIN1, DVL2, MSH6, EPAS1, VHL, RAF1, BIRC5, ITGA3, SMAD2, FZD3, FZD2, FZD5, RALGDS, STAT3, FZD6, WNT7B, CDKN1A, HDAC1, PLCG1, ITGA6, MTOR, IKBKB, CRK	0.0634205
Glioma	GRB2, TP53, RAF1, CDK6, PRKCG, PTEN, MAPK1, CCND1, CDKN1A, KRAS, PLCG1, CALM3, TGFA, MTOR, PIK3R3	0.0671433
Wnt signaling pathway	PPARD, MMP7, TCF7L2, PRKX, CSNK2A2, CSNK2A1, NFAT5, RHOA, NFATC2, FOSL1, PLCB2, AXIN1, DVL2, NLK, TP53, LEF1, PRKCG, SMAD2, FZD3, FZD2, FZD5, FZD6, CCND1, WNT7B, EP300, LRP6, SIAH1, WNT9A, TBL1X, TBL1Y	0.0697502
Melanogenesis	DVL2, CREB3, ADCY7, GNAI1, ADCY6, LEF1, RAF1, PRKCG, FZD3, FZD2, FZD5, TCF7L2, PRKX, FZD6, MAPK1, WNT7B, KRAS, EP300, CALM3, WNT9A, PLCB2	0.0781295
Non-small cell lung cancer	GRB2, ERBB2, TP53, RAF1, PRKCG, CDK6, MAPK1, CCND1, PDPK1, KRAS, PLCG1, TGFA, PIK3R3	0.0859618
Oxidative phosphorylation	UQCRC2, NDUFB4, COX11, ATP6V0E1, ATP6AP1, ATP6V1G1, ATP6V1B2, COX5A, ATP6V0B, NDUFS5, ATP6V0D1, ATP5H, NDUFS1, NDUFA2, NDUFA3, NDUFA8, COX4I1, LHPP, NDUFA10, NDUFA11, ATP6V1F, ATP6V1A, SDHB, UQCRH, ATP6V1E1, COX6A1	0.0863975
Huntington's disease	UQCRC2, NDUFB4, CLTB, POLR2I, CLTC, COX5A, NDUFS5, AP2B1, DNALI1, DLG4, TGM2, ATP5H, PLCB2, NDUFS1, HIP1, NDUFA2, NDUFA3, NDUFA8, SLC25A4, CREB3, TP53, COX4I1, NDUFA10, SOD1, VDAC2, POLR2J2, POLR2J3, SDHB, AP2A2, EP300, HDAC1, SP1, UQCRH, COX6A1, CLTCL1	0.0940157
Pancreatic cancer	ERBB2, TP53, RAF1, CDK6, SMAD2, STAT3, RALGDS, CDC42, ACVR1B, MAPK1, CCND1, KRAS, VEGFA, TGFA, IKBKB, PIK3R3	0.0944484
Protein export	OXA1L, SRP68, SRP19, SRP9	0.0986368

OE33OX4 versus OE33

OE33OX4 DAVID		
Term	Genes	p values
Vibrio cholerae infection	PRKCA, ACTB, KDELR3, ATP6V0E1, SLC12A2, ATP6AP1, PRKCG, ATP6V1G1, ATP6V1D, ATP6V0B, ATP6V1F, ATP6V1A, SEC61B, ATP6V0D1, SEC61A2	0.0036634
Lysosome	ARSB, AGA, CLN3, CLTA, CLTB, AP1G1, ATP6AP1, PPT2, AP3S1, ABCA2, CD63, ATP6V0B, AP1S3, LAMP1, CD68, NPC1, AP1S2, GLA, GALC, NEU1, CTSC, ATP6V0D1, CTSH, GBA	0.0067607
Phosphatidylinositol signaling system	PRKCA, IMPA2, PIK3CB, PIK3C2A, PIK3C2B, PIP5K1C, DGKH, PRKCG, ITPKB, DGKA, PLCD3, CALM3, INPP4A, INPP5D, PIP4K2A, PLCB2, PIP4K2C	0.0089694
Pathogenic Escherichia coli infection	PRKCA, ACTB, TUBB2A, ROCK2, CDH1, NCK2, CDC42, CTTN, EZR, MC1R, ARPC3, CLDN1, TUBB6, TUBA4A	0.0113136
Adherens junction	ACTB, PTPRJ, PTPRM, BAIAP2, WASF1, LMO7, ACTN1, CTNND1, CDH1, SMAD2, CTNNA1, ACP1, TCF7L2, SRC, CDC42, PVRL3, YES1	0.0131448
ErbB signaling pathway	PRKCA, PIK3CB, MAP2K2, CAMK2G, PRKCG, SRC, NRAS, NCK2, CDKN1A, PAK2, EREG, HBEGF, TGFA, MAPK8, NRG1, ABL2, MYC, SHC4	0.0194874
Fc gamma R-mediated phagocytosis	PRKCA, LIMK1, PIK3CB, MARCKSL1, WASF1, SPHK1, ASAP2, ASAP1, PIP5K1C, PRKCG, VAV2, PRKCD, CDC42, DOCK2, ARPC3, CFL2, SCIN, INPP5D, DNM1	0.0222315
Sphingolipid metabolism	SGPL1, ACER3, SPTLC1, GLA, SPTLC2, SPHK1, GALC, NEU1, SMPD4, GBA	0.0302417
Endocytosis	FGFR2, PRKCZ, CLTA, CLTB, RAB5B, CHMP5, ASAP2, PIP5K1C, ASAP1, SRC, CDC42, WWP1, GIT2, RAB11B, DNAJC6, STAM, TRAF6, VPS36, FAM125B, STAMBP, PSD3, RAB11FIP4, RAB11FIP5, RAB11FIP2, ADRB1, AP2A1, ACAP2, VPS24, GRK6, PDCD6IP, DNM1	0.0302968
Huntington's disease	POLR2H, CLTA, CLTB, POLR2L, POLR2I, COX7A2L, COX5B, UQCRQ, TFAM, NDUFS5, SIN3A, NDUFS8, ATP5O, PLCB2, HIP1, NDUFA2, NDUFA3, SLC25A4, CREB3, HTT, COX4I1, CREB5, POLR2J2, DCTN1, DCTN2, NDUFV3, HDAC2, SP1, AP2A1, COX6A1	0.0377116
Proteasome	PSMB5, PSMB4, PSMA6, PSME1, PSMC4, PSME2, PSMC1, PSMD2, PSMD6, PSMD7, PSMB8	0.0387551
Inositol phosphate metabolism	TPI1, IMPA2, PIK3C2A, PIK3CB, PIK3C2B, PLCD3, PIP5K1C, ITPKB, INPP4A, PIP4K2A, PLCB2, PIP4K2C	0.0414098
Epithelial cell signaling in Helicobacter pylori infection	ATP6V0E1, ATP6AP1, ATP6V1G1, CCL5, ATP6V1D, SRC, ATP6V0B, ATP6V1F, ATP6V1A, CDC42, HBEGF, MAPK8, IKBKB, ATP6V0D1	0.0446746
SNARE interactions in vesicular transport	STX5, STX4, STX1A, VAMP8, STX18, STX17, VTI1B, VAMP3, VAMP1	0.0644667
Pathways in cancer	FGFR2, PPARD, XIAP, MITF, FGF11, FOXO1, CDH1, FGF12, BCL2L1, TCF7L2, CDC42, CCNE1, MAX, LAMB3, LAMB2, PAX8, TGFA, FAS, FGF1, TRAF6, TRAF5, MYC, TRAF4, TRAF3, PRKCA, MSH6, BMP2, IL6, MAP2K2, PIK3CB, CDK6, PRKCG, FZD3, ITGA3, SMAD2, FZD5, STAT1, CTNNA1, STK4, DAPK3, STAT3, NRAS, CCDC6, CDKN1A, HDAC2, TCEB2, MAPK8, IKBKB	0.0645465
Oxidative phosphorylation	NDUFA2, ATP6V0E1, NDUFA3, ATP6AP1, COX4I1, ATP6V1G1, COX7A2L, UQCRQ, COX5B, ATP6V1D, ATP6V0B, PPA1, NDUFA11, ATP6V1F, NDUFV3, ATP6V1A, NDUFS5, NDUFS8, ATP5L, COX6A1, ATP5O, ATP6V0D1	0.0680354

OE33OX4 versus OE33 (continued)

OE33OX4 DAVID		
**Term**	**Genes**	**p values**
N-Glycan biosynthesis	MGAT4B, MGAT1, MGAT4A, ST6GAL1, TUSC3, DAD1, DPM2, ALG6, MAN1A1, RPN2	0.0764823
p53 signaling pathway	ZMAT3, CDK6, SFN, ATR, RRM2B, ATM, CCNE1, TP53I3, CDKN1A, TSC2, MDM4, FAS, THBS1	0.0867645
Insulin signaling pathway	PHKA2, PRKCZ, PIK3CB, MAP2K2, PHKG2, PHKA1, FLOT1, SOCS1, PRKAB1, FOXO1, PPP1CB, NRAS, PPP1R3C, PYGL, TSC2, PRKAR1A, CALM3, MAPK8, INPP5D, IKBKB, TRIP10, SHC4	0.0933216
Small cell lung cancer	XIAP, PIK3CB, CDK6, ITGA3, BCL2L1, MAX, CCNE1, LAMB3, LAMB2, TRAF6, IKBKB, TRAF5, MYC, TRAF4, TRAF3	0.0991423

OE21OX4 versus OE21

OE21OX4 DAVID		
**Term**	**Genes**	**p values**
Lysosome	AGA, CTSL2, NPC1, SLC17A5, PSAP, IGF2R, CTSD, ACP2, CTSA, ATP6V0D1, ATP6V0B, CTSL1	0.0202848
Glioma	PRKCA, E2F2, IGF1R, CAMK2G, ARAF, TP53, TGFA, CALM1	0.0267008
Prion diseases	NOTCH1, LAMC1, HSPA5, PRNP, CCL5	0.0787785
Pathways in cancer	PRKCA, E2F2, DVL3, BMP2, AR, STK36, CBL, TP53, ITGB1, MMP2, CTNNB1, FOS, IGF1R, WNT7B, LAMA3, LAMB2, LAMA5, ARAF, VEGFA, TGFA, LAMC2, LAMC1	0.0855068
Tight junction	PRKCA, EPB41L2, CLDN7, RAB3B, EPB41L1, CLDN4, CSNK2B, EXOC3, AMOTL1, PRKCE, CTNNB1	0.0959217
Small cell lung cancer	E2F2, LAMA3, LAMB2, LAMA5, TP53, LAMC2, LAMC1, ITGB1	0.0968986
